# Comparison of alterations in local field potentials and neuronal firing in mouse M1 and CA1 associated with central fatigue induced by high-intensity interval training and moderate-intensity continuous training

**DOI:** 10.3389/fnins.2024.1428901

**Published:** 2024-08-15

**Authors:** Yuncheng Liu, Weiyi Lao, Haojie Mao, Yaoyao Zhong, Jihui Wang, Wei Ouyang

**Affiliations:** College of Physical Education and Health Sciences, Zhejiang Normal University, Jinhua, China

**Keywords:** exhausting exercise, primary motor cortex, hippocampal CA1, local field potentials, neuronal firing

## Abstract

**Background:**

The mechanisms underlying central fatigue (CF) induced by high-intensity interval training (HIIT) and moderate-intensity continuous training (MICT) are still not fully understood.

**Methods:**

In order to explore the effects of these exercises on the functioning of cortical and subcortical neural networks, this study investigated the effects of HIIT and MICT on local field potential (LFP) and neuronal firing in the mouse primary motor cortex (M1) and hippocampal CA1 areas. HIIT and MICT were performed on C57BL/6 mice, and simultaneous multichannel recordings were conducted in the M1 motor cortex and CA1 hippocampal region.

**Results:**

A range of responses were elicited, including a decrease in coherence values of LFP rhythms in both areas, and an increase in slow and a decrease in fast power spectral density (PSD, *n* = 7–9) respectively. HIIT/MICT also decreased the gravity frequency (GF, *n* = 7–9) in M1 and CA1. Both exercises decreased overall firing rates, increased time lag of firing, declined burst firing rates and the number of spikes in burst, and reduced burst duration (BD) in M1 and CA1 (*n* = 7–9). While several neuronal firing properties showed a recovery tendency, the alterations of LFP parameters were more sustained during the 10-min post-HIIT/MICT period. MICT appeared to be more effective than HIIT in affecting LFP parameters, neuronal firing rate, and burst firing properties, particularly in CA1. Both exercises significantly affected neural network activities and local neuronal firing in M1 and CA1, with MICT associated with a more substantial and consistent suppression of functional integration between M1 and CA1.

**Conclusion:**

Our study provides valuable insights into the neural mechanisms involved in exercise-induced central fatigue by examining the changes in functional connectivity and coordination between the M1 and CA1 regions. These findings may assist individuals engaged in exercise in optimizing their exercise intensity and timing to enhance performance and prevent excessive fatigue. Additionally, the findings may have clinical implications for the development of interventions aimed at managing conditions related to exercise-induced fatigue.

## Introduction

Human exercise fatigue is a multifaceted physiological state associated with decreased physical performance, and can arise from a combination of CF and peripheral fatigue (PF) (Weavil and Amann, 2019; Tornero-Aguilera et al., 2022). CF is characterized by complex interactions between cortical and subcortical neural networks and deficient motor cortical output, whereas PF causes impaired neuromuscular junction and/or muscle function (Weavil and Amann, 2019; Tornero-Aguilera et al., 2022). However, it is conceivable that human CF and PF may occur asynchronously, and the underlying mechanisms, especially those governing CF, remain elusive and incompletely elucidated (Carroll et al., 2017; Burnley and Jones, 2018). The relative contributions of CF and PF to exercise fatigue may vary depending on factors such as exercise intensity and duration, gender, sport type, as well as emotional and psychological factors (Burnley and Jones, 2018; Tornero-Aguilera et al., 2022). However, the precise mechanisms underlying exercise fatigue in both HIIT and MICT remain incompletely understood.

The outcomes derived from both animal models (Khodagholy et al., 2017; Nitzan et al., 2020) and human (Kunz et al., 2024) studies reveal a functional connectivity between the hippocampus and the cortex, mediated by the entorhinal region and the default mode network, via analogous ripple oscillations. This interconnection underscores the intricate interplay among diverse brain regions and networks during cognitive, psychological, and physiological functions. Despite this, there remains a paucity of knowledge regarding the precise flow of information between the human hippocampus and the cortex (Smallwood et al., 2021). Extensive human research has delineated the pivotal role of the hippocampus in the consolidation of motor sequence memory (Albouy et al., 2008; Jacobacci et al., 2020). This consolidation process is further correlated with a notable augmentation in blood perfusion within the hippocampus, indicating its involvement in sequence memory formation and retention (Fernández-Seara et al., 2009).

Long-term aerobic exercise (Voss et al., 2010) and MICT (Weng et al., 2017) could enhance human hippocampal-cortical connectivity. Although various modalities of moderate-to-high-intensity exercise have exhibited beneficial effects on human brain health, encompassing improvements in mental wellbeing, augmentation of hippocampal volume, and enhancements in spatial and verbal learning capabilities (Gorham et al., 2019; Rodriguez-Ayllon et al., 2019; Prathap et al., 2021), excessive exercise-induced fatigue can negatively impact cognitive performance. Notably, human studies have revealed that high-intensity exercise leading to the onset of CF significantly prolongs the response time of individuals during memory-demanding vigilance tests (Moore et al., 2012) and adversely affects their performance in complex cognitive tasks (Anders et al., 2021). Severe and/or prolonged CF has been associated with an array of negative effects, including depression, pain, sensations of exhaustion, challenges in maintaining cognitive vigilance, and difficulties in sustaining mental attention (Leavitt and DeLuca, 2010). However, CF has also been proposed to serve as a protective mechanism for human body during maximal or endurance exercise, guarding against potential damage (Noakes, 2012). Additionally, the structural and neural circuit alterations that have been described previously may contribute to human cognitive deficits, such as reduced cognitive flexibility to external stimuli, difficulties in goal-directed cognition, and impairments in memory function (Price and Duman, 2020).

Despite the substantial evidence from both human and animal research highlighting the positive effects of exercise on neuroplasticity, cognition, mood, and prevention of neurodegenerative disorders, mediated by the upregulation of neurotrophic factors (De Sousa Fernandes et al., 2020), attenuation of inflammation and protein misfolding, as well as the preservation of synaptic integrity (Sujkowski et al., 2022), a notable gap in knowledge persists regarding the intricate mechanisms linking excessive exercise-induced fatigue to alterations in brain neural network function. This deficiency is primarily due to a scarcity of animal model studies that explore this relationship (Yan et al., 2022), necessitating further research to enhance our comprehensive understanding of this critical field.

The intracerebral LFP in humans or animals serves as a metric for neural activity, recorded at the aggregated level of bioelectrical signals. Notably, the vast majority of LFP activity originates from non-local, remote sources, thereby reflecting the dynamic information flow across intricate neural networks (Herreras, 2016). LFP is intricately linked to diverse behavioral and cognitive processes, each associated with specific brain regions and oscillatory dynamics. Consequently, LFP has garnered substantial attention in both scientific research and clinical diagnosis (Ibarra-Lecue et al., 2022). By scrutinizing neuronal firing patterns, we can elucidate the localized impact of oscillations on the temporal synchronization of neuronal spikes. Furthermore, analyzing the correlation between firing rates and oscillations in long-range neural circuits offers compelling insights into the genesis of oscillatory rhythms, particularly by identifying neuronal populations that exhibit spontaneous firing at distinct frequencies and generating oscillations under diverse conditions.

Characterizing neural dynamics and neuronal firing patterns can aid in elucidating the mechanisms underlying exercise-induced CF. To this end, we designed an experimental protocol involving exhaustive HIIT and MICT in a mouse model. We will subsequently assess the alterations in LFP and neuronal firing in M1 and CA1 following HIIT/MICT. Our findings aim to provide novel insights into the neural compensatory and self-protective mechanisms activated during CF, thereby contributing to the optimization of exercise performance, minimization of excessive fatigue, and the development of targeted therapeutic interventions for clinical conditions associated with exercise-induced fatigue.

## Materials and methods

### Animals

Male C57BL/6J mice (18–20 g, 8–10-week old, Experimental Animal Center, Zhejiang Academy of Medical Science) were housed under 12-h light-dark cycles and had free access to food and water throughout the study. All procedures involved in this study were performed under the Institutional (Zhejiang Normal University, the ethical approval number: ZSDW2022032) Animal Care and Use Committee approval. All methods were performed also in accordance with the ARRIVE guidelines and the NIH Guide for the Care and Use of Laboratory Animals (2011 edition). Mice were randomly divided into three groups: Control (CON, *n* = 9), HIIT (*n* = 7), and MICT (*n* = 9). The mice were habituated for 5 days in the laboratory environment before electrode implantation surgery.

### Intra-motor cortex and CA1 recording and analysis

In the pre- and post-exercise stages of this study, spontaneous neuronal firing and LFP were recorded simultaneously in both the M1 and CA1 regions. After administering anesthesia (sodium pentobarbital, 45 mg/kg i.p), two Teflon-coated bipolar microelectrodes (0.002-inch nichrome wire, spaced 300 μm apart; A-M Systems, Carlsborg, WA) were inserted into the right hemisphere of the mouse brain based on the mouse brain atlas (*The Mouse Brain in Stereotaxic Coordinates, Paxinos G, Franklin KBJ, fifth edition*), targeting the M1 region (AP + 1.8 mm, ML + 1.6 mm, DV – 1.0 mm from skull surface) and the CA1 region (AP – 2.0 mm, ML + 1.4 mm, DV – 1.9 mm from skull surface) through a 1.5–2 mm diameter hole. Ground electrodes were placed on the skull near the Lambda suture. A small amount of a mixture of mineral oil and bone wax was packed around all the electrode penetration zones. One support screw was placed over the frontal part of the skull, and the whole ensemble was secured with dental cement. Mice were transferred to an independent cage after surgery. For acclimation purpose, mice were connected with a flexible tethered cable to the signal acquisition system (BL-420 Biological Function Experimental System, Chengdu Taimeng Company, Chengdu, China). Mice were carefully watched for any adverse reactions and allowed 3 days recovery before initiating adaptive treadmill training.

**Figure 1:**
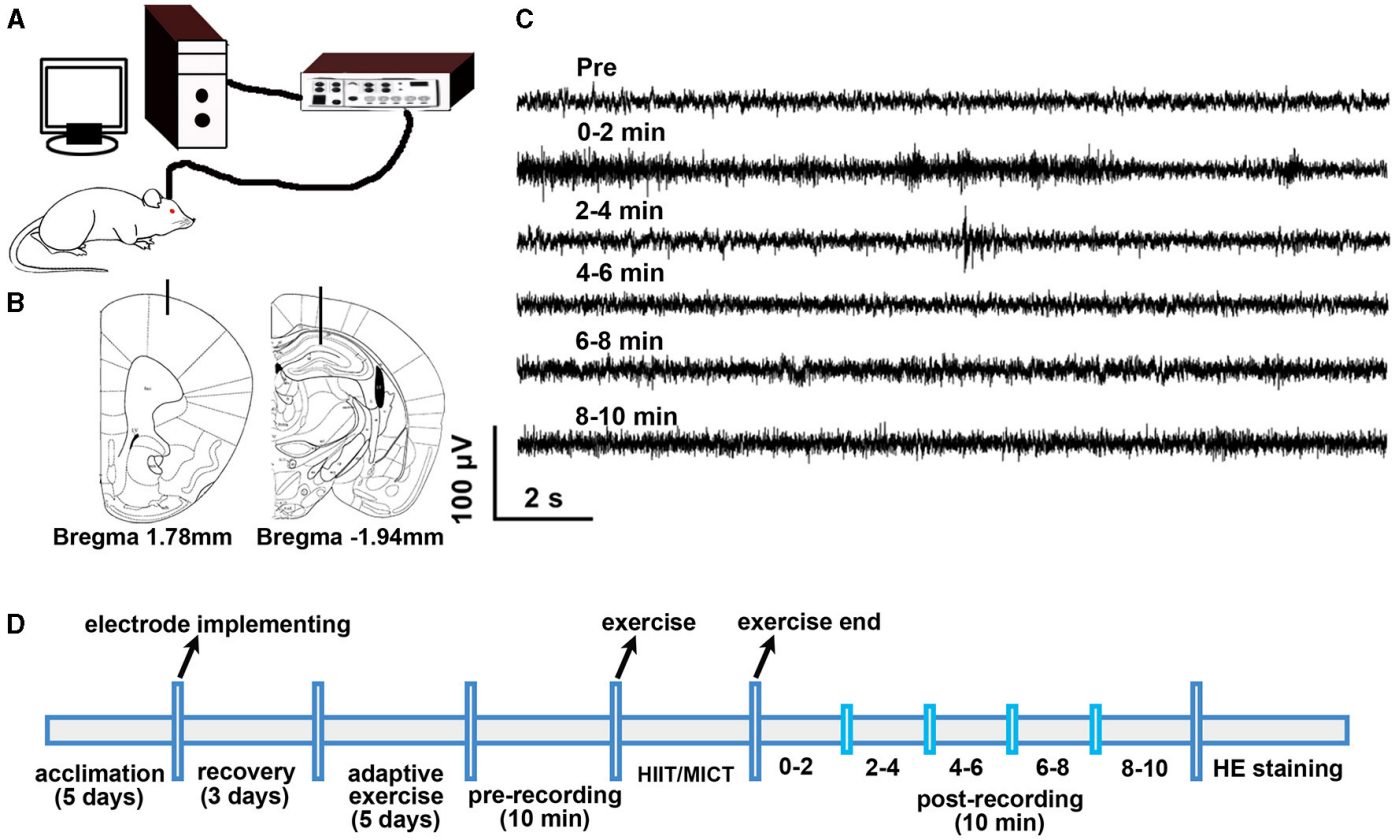
Experimental design. **(A)** Electrophysiological recording setup. **(B)** Electrode location in M1 and CA1. **(C)** Sample of raw electrophysiological data. **(D)** Experimental timeline.

Simultaneous extracellular recordings were made from the M1 and CA1 for 10 min pre- and post- HIIT/MICT (Figure 1) while the mice move freely in the open bucket (diameter: 20 cm; height: 40 cm). The recorded raw neural signals are composed of two main components: LFP and spikes (neuronal firings). The recorded raw signals from the electrode were amplified and band-pass filtered from 0.16–3 kHz, and the sampling frequency was set at 10 K Hz. The notch filter of the amplifiers was kept on to eliminate 50 Hz interference. The firing data was output as a txt file and analyzed using Spike2 (CED, Cambridge, UK). LFP measures the compound synaptic activity-generated by a large pool of neurons, which is especially helpful for the study of long-range interactions. The LFP data were obtained by down-sampling the raw data with a sampling rate of 500 Hz and 2nd order Butterworth band filter of 1.3–80 Hz (Ahmadi et al., 2021; Orellana et al., 2024), in contrast to the typical 300 Hz low-pass cut-off frequency. This protocol was implemented to eliminate potential contamination from nearby multiunit spiking activity within higher LFP band (100–300 Hz) (Ahmadi et al., 2021) and animal slow movement (Orellana et al., 2024).

To prevent Spike2 software from struggling when analyzing large chunks of raw data, only the last 2 min recording data during pre-exercise recording was adopted as pre-HIIT/MICT baseline, and post-HIIT/MICT 10 min data was evenly divided into five subgroups (0–2, 2–4, 4–6, 6–8, 8–10 min) for a more detailed analysis of the transition in LFP and neuronal firing properties. The power values within specified frequency bands (delta 1.5–4 Hz, theta 4–10 Hz, alpha 10–13 Hz, beta 13–30 Hz, gamma 30–80 Hz) (Einevoll et al., 2013; Crouch et al., 2018; Jonak et al., 2018) were averaged from every 2 min epoch of the 10 min LFP raw data with 0.39 Hz frequency resolution (Figure 1). Since it took ~2–3 min to transfer the mouse from the recording bucket to the treadmill, and vice versa, a 5-min interval was introduced between the pre-exercise recording and the HIIT/MICT, as well as between the HIIT/MICT and post-exercise recording. This 5-min buffer period was also useful for minimizing the potential effects of stress and fear induced by the exercise on electrophysiological recording.

Every raw data was visually examined for abnormal electrographic activity, possibly due to movement, ECG, etc. As high-frequency components of LFPs can be generated up to ~500 Hz (Einevoll et al., 2013), overall neuronal firing data were extracted from raw neural signal by implementing a 2nd order Butterworth high-pass filter with a 500 Hz cut-off frequency and down-sampled to 2 kHz using Spike 2 software (Singh et al., 2023). The baseline drift was adjusted by running HUM REMOVE script in Spike 2. Moreover, we also run channel processing function to remove direct current component and rectify the large slopes. Spike sorting was carried out by first selecting a threshold baseline and running the Spike 2 spike sorting function. This produced a full wave template that was then used to discriminate multi-unit spikes using principal component analysis (PCA) clustering. The neuronal firing frequency in a recorded channel of M1 or CA1 was analyzed by running IF HIST script in Spike2.

In this study, to evaluate the alterations of synchronization in M1 and CA1 among different group mice, the general coherence of extracellular firing patterns in M1 and CA1 were calculated by running COHERENCE script [$coh\;\left(f\right)\;=\;|\sum csd_{ab}\left(f\right){|}^{2}/\sum psd_{a}\left(f\right)\sum psd_{b}\left(f\right)$, csd, current source density; psd, power spectral density] in Spike2 software.

Legendy and Salcman assumed that the frequency of spike trains had a Poisson distribution, and defined bursts of spikes by the value of the “Poisson surprise” parameter (Legendy and Salcman, 1985). A surprise value (negative logarithmic transform of the probability), measures how unlikely a cluster of spikes within a time interval. A burst is defined as at least three consecutive spikes with two sequential ISI (inter-spike interval) less than one-half of the mean ISI of all spikes. Bursts with Surprise values >10 were used to analyze the burst per 2 min, the normalized burst rate relative to the overall firing rate, spikes in burst and BD. CROSS CHANNEL CORRELATION script in Spike 2 software was used to analysis the temporal and/or spatial relationships between M1 and CA1 spike trains.

GF reflects the density distribution of high-frequency components and power spectral entropy (PSE) in the spectrum. GF alteration describes the migration of the gravity center in the power spectrum (Li H. et al., 2019). PSE represents the irregularity in a signal's power spectrum (Frohlich et al., 2021). GF has a strong correlation with mental fatigue (Li H. et al., 2019), while PSE is positively correlated with consciousness level (Frohlich et al., 2021). Acute and prolonged exercise also cause physical and mental fatigue in mice (Xu et al., 2017; Lee et al., 2022). To analyze the alteration of GF, the 1.3–80 Hz LFP data obtained using Spike2 were exported as txt files; subsequently, the last 2 min of pre-exercise and every 2 min of the 10 min post -HIIT/MICT LFP data were imported into Matlab (v2016, Mathworks, Natick, MA). The GF analysis was done by running a home-made gravity-frequency script [$f_{g}\;=\;\overset{f_{2}}{\underset{f\;=\;f_{1}}{\sum }}\left(psd\left(f\right)\times f\right)/\overset{f_{2}}{\underset{f\;=\;f_{1}}{\sum }}psd\left(f\right)$, *f*_g_ is the GF; *f*_1_ to *f*_2_, the lower limit and higher limit on the total band; psd, power spectral density] in MATLAB. The schematic diagram of electrophysiological recording is shown in Figure 1A. The implantation locations of electrodes in M1 and CA1, and sample of raw data are shown in Figures 1B, C respectively. The experimental timeline is shown in Figure 1D. To confirm the accuracy of electrode placement, the immunohistochemical staining method, as previously described in our prior work (Ouyang et al., 2017), was utilized to visualize the electrode path in mouse brains 1–2 days after electrophysiological recording.

### HIIT and MICT

The HIIT/MICT group mice were allowed adaptive treadmill (ZH-PT/5S Rodent Treadmill, Anhui Zhenghua Biological Apparatus Company, Huaibei, China) training for 5 days before formal HIIT and MICT training. The active time of C57 mice was 6–12 pm daily, and their daily running distance was mostly completed during this period (Taylor et al., 2015). The peak activity period was between 6–8 pm and the average running speed of C57 mice was ~11 m/min (Taylor et al., 2015). According to a previous study, this running speed was approximately the exercise intensity of ~50% VO_2_max (Baker and Gleeson, 1999). This amount of exercise is equivalent to human low-intermediate intensity aerobic exercise. Accordingly, we chose to perform mouse treadmill HIIT or MICT and extracellular recording between 6 and 8 pm.

During adaptive treadmill training, all mice underwent the same training protocol. Specifically, mice run on a treadmill at 5 m/min, 20 min on day 1; 8 m/min, 20 min on day 2; 11 m/min, 30 min on day 3; 16 m/min, 5 min on day 4; 21 m/min, 1 min on day 5. HIIT and MICT procedure was adapted from previously published training protocol in which the same strain mouse was used (Li B. et al., 2019). C57 mice can run at a maximum speed of 24.30 ± 0.87 m/min (Li B. et al., 2019). The HIIT includes 10 cycles, each cycle includes 21 m/min, 1.2 min high-speed running and 11 m/min, 2 min low-speed running, while the MICT comprises 15 m/min running at a constant speed for 32 min (Figure 1D). Mice were motivated to run on the treadmill until they were incompetent or unwilling to continue, in an effort to escape further electric shocks delivered in a pulsatile-mode lasting 200 ms at a frequency of 2 Hz and amplitude of 1.22 mA (Dougherty et al., 2016). These electric shocks were comparable to a mild tingling sensation when touched by an ungloved finger and did not produce severe discomfort. To confirm the mice had reached a state of physiologic exhaustion, the criterion for exhaustion was defined as remaining on the shock grid for at least five consecutive seconds and failing to resume running despite repeated aversive stimuli (Dougherty et al., 2016). To avoid environmental bias among sedentary control mice not undergoing the exercise protocol, mice were transferred and housed in a separate room during exercise sessions. Sedentary control mice were confined to lanes on a stationary treadmill for 10 min (Seldeen et al., 2019).

### Statistical analysis

The parameters of electrophysiological data are expressed as the mean ± standard deviation (SD). All data were tested for normal distribution using Kolmogorov and Smirnov test (GraphPad Prism 9, San Diego, CA). Normally distributed data were analyzed using one-way ANOVA followed by *Bonferroni* multiple *post hoc* comparisons or Brown-Forsythe and Welch ANOVA followed by *Dunnett* multiple *post hoc* comparisons using Prism 9. Statistically significant differences were denoted by *p* values, such as ^*^p < 0.05; ^**^p < 0.01 and ^***^p < 0.001 vs. control values; ^†^*p* < 0.05; ^††^*p* < 0.01 vs. MICT values. ^##^*p* < 0.01 vs. pre-exercise values; ^&^*p* < 0.05; ^&&^*p* < 0.01 compare to the values among other different time slots post-exercise.

## Results

### Mice underwent HIIT and MICT showed fatigue features

In the early stage of the exercise, the mice ran in a good cadence, and could complete the predetermined intensity running. When HIIT is carried out to the 5th cycle or MICT to 23 min, the mice started to show signs of weariness (running speed occasionally falling behind the predefined treadmill speed, and sometimes pause on the running belt), but could still maintain the predetermined intensity running by poking, or touching them with a hand (Dougherty et al., 2016). After HIIT proceeded into the 8–9th cycle or MICT to 30–32 min, the mice kept running behind the predefined speed. When the mouse showed above mentioned fatigue-like behavior and exhaustion criterion, the treadmill exercise ended. At the end of the HIIT or MICT protocol, all mice meet the criterion for exhaustion.

### The effects of HIIT/MICT on LFP correlation in M1 and CA1

**Figure 2:**
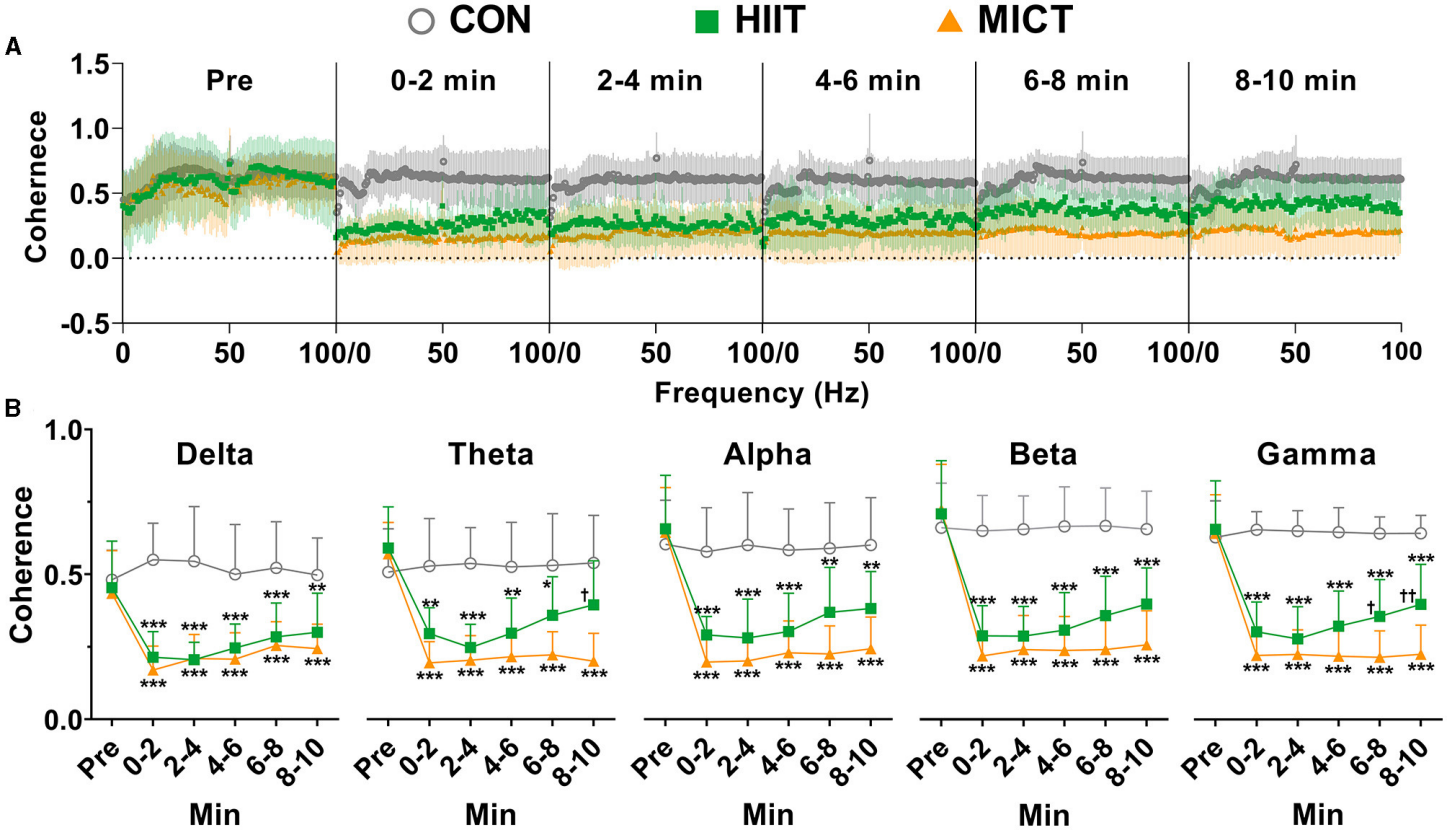
The coherence values between LFP in M1 and CA1 post-HIIT/MICT. **(A)** Coherence alterations of slow and fast LFP rhythms in M1 and CA1 post HIIT/MICT. **(B)** The coherence changes in each slow and fast LFP rhythm in M1 and CA1 post-HIIT/MICT were statistically summarized. The data in the figure is represented by mean ± SD, *n* = 7–9. *p < 0.05, **p < 0.01, ***p < 0.001 vs. control values; ^†^*p* < 0.05, ^††^*p* < 0.01 vs. MICT values by Brown-Forsythe and Welch ANOVA followed by *Dunnett* multiple *post hoc* comparisons.

To understand the temporal correlation between activities at M1 and CA regions, we simultaneously recorded LFP at the two sites. During pre-exercise phase, the LFP rhythm in M1 and CA1 showed a consistently increasing trend of temporal correlation from slow to fast oscillation, manifested by increasing coherence values (delta:0.44 ± 0.15; theta: 0.58 ± 0.13; alpha:0.65 ± 0.17; beta: 0.72 ± 0.17; gamma: 0.65 ± 0.15, *n* = 16). Beta activity in M1 and CA1 displayed the highest correlation. To demonstrate the temporal correlation of activities between M1 and CA1, coherence values were averaged across each 2-min slot during the pre-exercise and 10-min post-HIIT/MICT period. These values are presented in Figure 2A.

The coherence values of theta (0–2 min, *p* < 0.05; 2–4 min, *p* < 0.01; 4–6 min, *p* < 0.05), alpha (0–2, 2–4, and 4–6 min, *p* < 0.05), beta (0–2, 2–4, 4–6, and 6–8 min, *p* < 0.05), and gamma (0–2, 2–4, and 4–6 min, *p* < 0.05) rhythms in both M1 and CA1 consistently decreased post-HIIT, as compared to pre-exercise values, and were statistically analyzed by Brown-Forsythe and Welch ANOVA (Figure 2B). Notably, the coherence values of delta (0–2 min, *p* < 0.05), theta (0–2, 2–4, 6–8 and 8–10 min, *p* < 0.001), alpha (0–2, 2–4, 6–8 and 8–10 min, *p* < 0.001), beta (0–2, 2–4, 6–8 and 8–10 min, *p* < 0.001), gamma (0–2, 2–4, 6–8 and 8–10 min, *p* < 0.001) rhythms in M1 and CA1 consistently but more significantly decreased post-MICT, as compared to pre-exercise values, and statistically analyzed by same method (Figure 2B).

Compared with the counterpart control values, MICT also displayed more potent effects of suppressing the coherency cross slow and fast rhythms during 10 min post exercise (Figure 2), while the coherence values of all slow and fast rhythms post-HIIT showed clear recovery tendency (Figure 2). These results indicate that both HIIT and MICT can hamper the temporal correlations of activities between M1 and CA1, thus the functional integration of neural circuits was altered. Compared to HIIT fatigue, MICT fatigue is associated with more substantial and steadier suppression of functional integration between M1 and CA1, and typically on theta, alpha, beta and gamma rhythms (Figure 2). These results may help to explain the more profound CF induced by MICT vs. HIIT.

### The effects of HIIT/MICT on slow LFP rhythms

**Figure 3:**
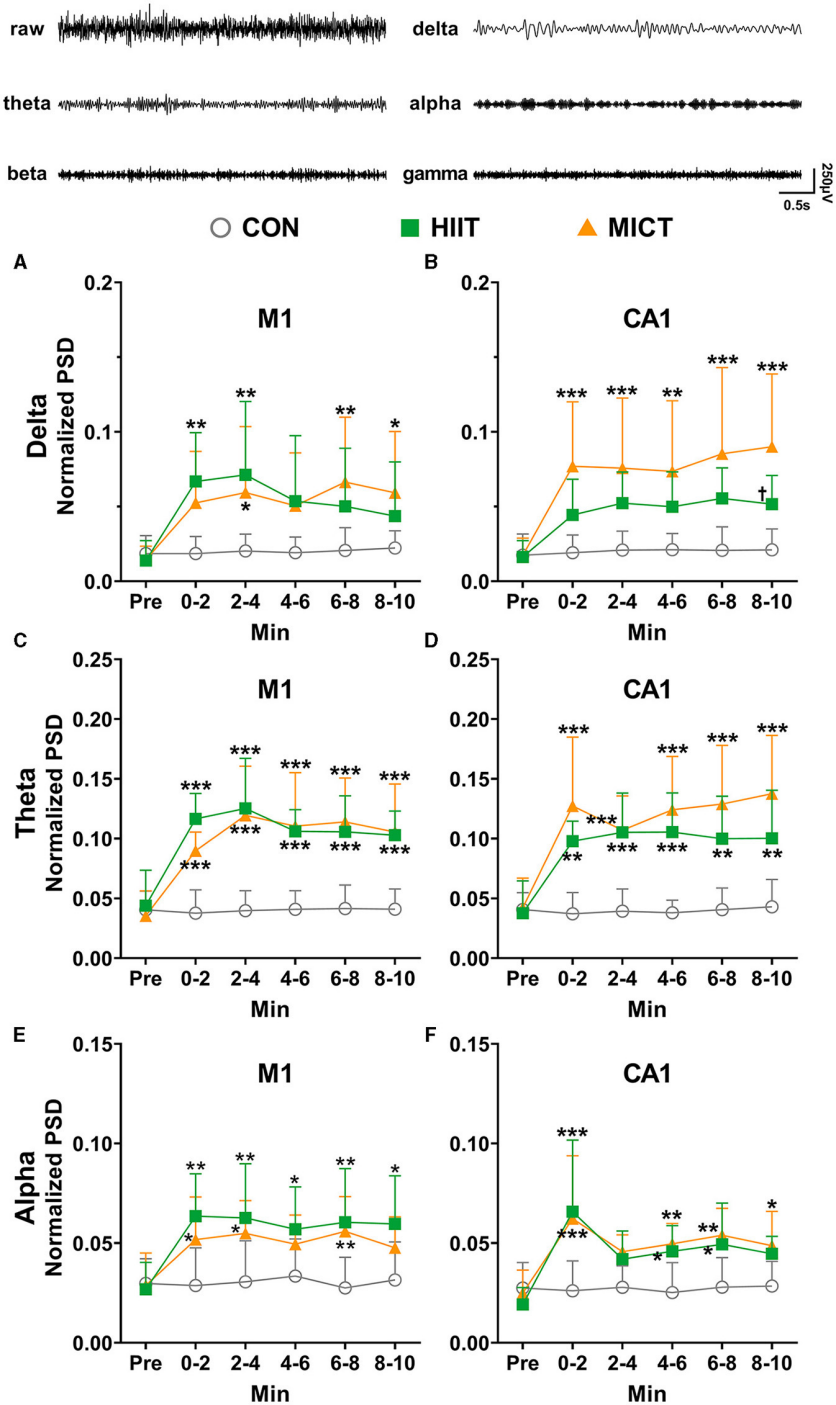
The normalized LFP slow rhythmic PSD alterations post-HIIT/MICT. The represent traces of delta, theta, alpha, beta and gamma are shown on the top panel. **(A, B)** Delta power changes in M1 and CA1 post-HIIT/MICT; **(C, D)** Theta power changes in M1 and CA1 post-HIIT/MICT; **(E, F)** Alpha power changes in M1 and CA1 post-HIIT/MICT. The data is represented by mean ± SD, *n* = 7–9. *p < 0.05, **p < 0.01 vs. control values; ^†^*p* < 0.05 vs. MICT values by Brown-Forsythe and Welch ANOVA followed by *Dunnett* multiple *post hoc* comparisons. ***p < 0.001 vs. control values.

In order to eliminate the effects of the individual variation, the band powers (delta, theta, alpha, beta, gamma, the represent traces are shown on the top panel of Figure 3) were normalized to the total power. The predominant alterations of PSD occurred in a slow frequency range (delta, theta and alpha, Figure 3). Compared to the pre-exercise values, the PSDs of delta (Figures 3A, B), theta (Figures 3C, D) and alpha (Figures 3E, F) consistently increased in M1 (theta: 0–2 min, *p* < 0.01; 2–4, 4–6, 6–8 and 8–10 min, *p* < 0.05 by Brown-Forsythe and Welch ANOVA, Figure 3C) and CA1 (delta: 6–8 and 8–10 min, *p* < 0.05; theta: 0–2, 2–4 and 4–6 min, *p* < 0.05; alpha: 4–6 min, *p* < 0.05; 6–8 min, *p* < 0.01 by Brown-Forsythe and Welch ANOVA, Figures 3B, D, F) during each 2-min time slot following HIIT. Similarly, consistently increased in M1 (theta: 0–2 min, *p* < 0.001, 2–4 min, *p* < 0.01, 4–6 min, *p* < 0.05, 6–8 min, *p* < 0.01, and 8–10 min, *p* < 0.05 by Brown-Forsythe and Welch ANOVA, Figure 3C) and CA1 (delta: 8–10 min, *p* < 0.01 by one-way ANOVA; theta: 0–2 min, *p* < 0.05, 2–4 and 4–6 min, *p* < 0.01, 6–8 min, *p* < 0.05, and 8–10 min, *p* < 0.01; alpha: 2–4 min, *p* < 0.05, 4–6 and 6–8 min, *p* < 0.01 by Brown-Forsythe and Welch ANOVA, Figures 3B, D, F) during each 2-min time slot post-MICT. When compared with the control values, all slow rhythms, typically delta and theta PSDs steadily increased in both M1 and CA1 during each 2 min slots following HIIT/MICT. Notably, greater increases in delta (8–10 min, *p* < 0.05 vs. HIIT value, Figure 3B) and theta (Figure 3D) PSDs were observed in CA1 post-MICT compared to the corresponding values following HIIT.

Pearson's correlation analysis showed that the increased PSD of delta (*r* = 0.779, *p* = 0.039), theta (*r* = 0.958, *p* < 0.001) and alpha (*r* = 0.861, *p* = 0.013) in M1 significantly correlated with the parallel values in CA1 after HIIT. Similarly, the increased PSD of delta (*r* = 0.889, *p* = 0.002), theta (*r* = 0.857, *p* = 0.003) and alpha (*r* = 0.854, *p* = 0.002) in M1 significantly correlated with the matching values in CA1 after MICT. Thus, both M1 and CA1 displayed synchronically increased PSD of slow LFP oscillation after HIIT/MICT. Therefore, both HIIT and MICT can slow down the local neural network function and/or neural circuit activity between M1 and CA1. These results parallel previous coherence results and suggest the inhibition of the functional integration of neural circuits between M1 and CA1.

Pearson's correlation analysis showed that delta (*r* = 0.777, *p* = 0.039), theta (*r* = 0.914, *p* = 0.004) and alpha (*r* = 0.962, *p* < 0.001) PSD increasing trend in M1 are significantly correlated post HIIT/MICT. Similarly, delta (*r* = 0.882, *p* = 0.009), theta (*r* = 0.913, *p* = 0.004) and alpha (*r* = 0.988, *p* < 0.001) PSD increasing trend in CA1 are also significantly correlated post HIIT/MICT. Accordingly, slow LFP rhythms showed similar increasing patterns in M1 or CA1 after HIIT/MICT. Compared to the potent effects of HIIT and MICT on PSD of slow LFP, MICT fatigue is associated with a clearer increase of delta PSD and an increasing trend of theta PSD in CA1. Delta and theta were more prominently associated with MICT fatigue (Figure 3).

### The effects of HIIT/MICT on fast LFP rhythms

**Figure 4:**
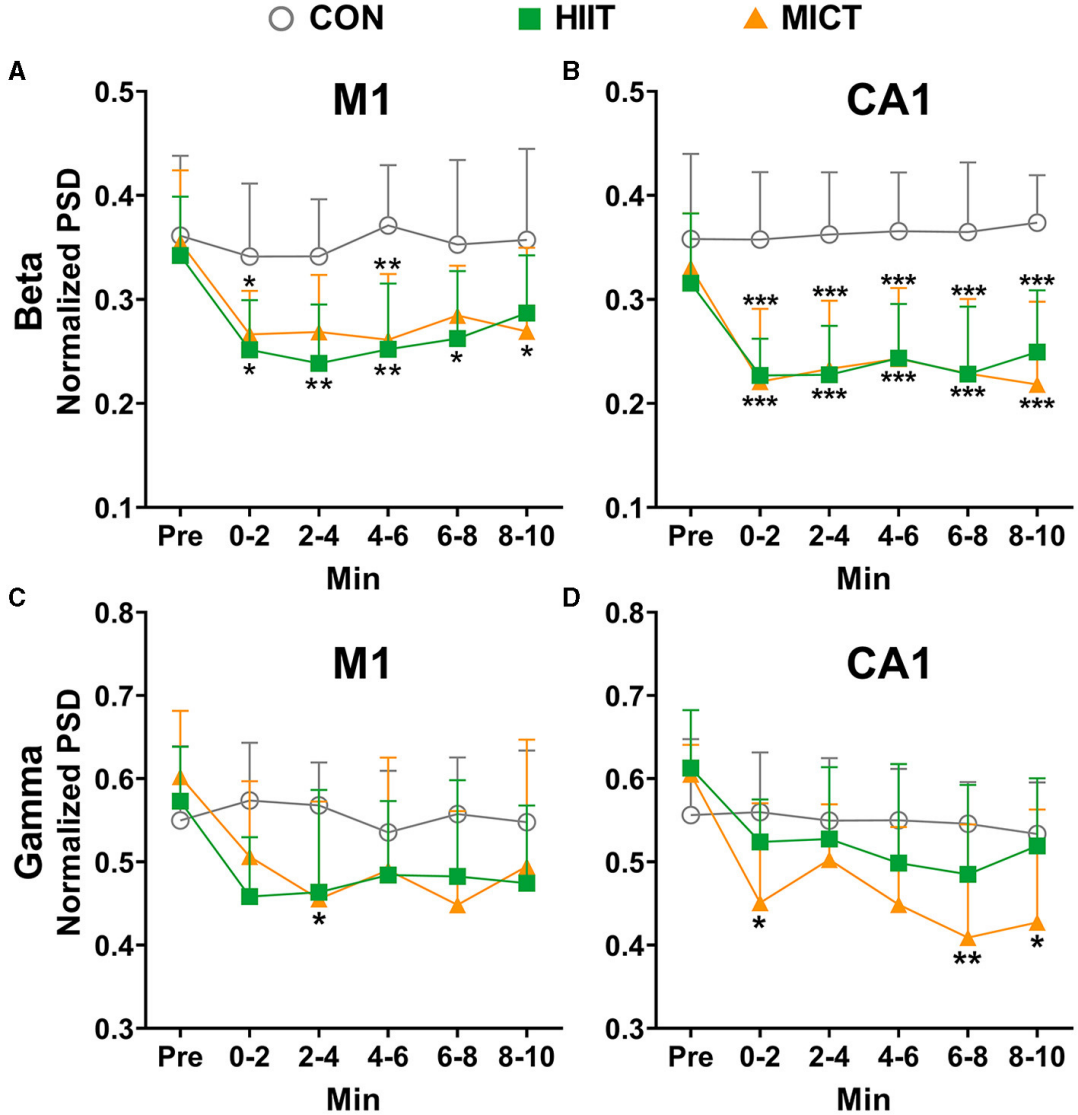
The normalized LFP fast rhythmic power spectrum alterations post-HIIT/MICT. **(A, B)** Beta power changes in M1 and CA1 post- HIIT/MICT; **(C, D)** Gamma power changes in M1 and CA1 post- HIIT/MICT. The data is represented by mean ± SD, *n* = 7–9. *p < 0.05 vs. control values by Brown-Forsythe and Welch ANOVA followed by *Dunnett* multiple *post hoc* comparisons. **p < 0.01, ***p < 0.001 vs. control values.

Beta and gamma are typical rhythms associated with active behaviors (Zheng and Colgin, 2015). When compared to the pre-exercise or control values, the PSDs of beta (Figures 4A, B) and gamma (Figures 4C, D) consistently decreased in M1 and CA1 during each 2-min time slot post HIIT. Similarly, the beta (Figures 4A, B) and gamma (Figures 4C, D) PSDs consistently decreased in M1 and CA1 (beta: 0–2 min, *p* < 0.05; Gamma: 2–4, 4–6 and 6–8 min, *p* < 0.05 vs. pre-MICT values by Brown-Forsythe and Welch ANOVA, Figures 4B, D) during post MICT. Notably, CA1 exhibited a more consistent decrease in beta PSD and a stronger tendency toward decreased gamma PSD compared to M1, particularly after MICT (Figure 4).

Pearson's correlation analysis showed that the decreasing trend of beta (*r* = 0.951, *p* < 0.001) and gamma (*r* = 0.827, *p* = 0.022) in M1 significantly correlated with the parallel values in CA1 after HIIT. Similarly, the reduced PSD of beta (*r* = 0.941, *p* < 0.001) and gamma (*r* = 0.802, *p* = 0.009) in M1 significantly correlated with the matching values in CA1 after MICT. Thus, besides the aforementioned enhanced slow rhythm activity post-HIIT/MICT, the results further indicate the inhibition of the functional neural circuit activities between M1 and CA1, and suppressed fast rhythms such as beta and gamma both in M1 and CA1 are linked to HIIT/MICT fatigue.

Pearson's correlation analysis showed that beta (*r* = 0.904, *p* = 0.005) and gamma (*r* = 0.856, *p* = 0.014) PSD decreasing trend in M1 are significantly correlated post-HIIT/MICT. Similarly, beta (*r* = 0.939, *p* = 0.002) and gamma (*r* = 0.947, *p* = 0.001) PSD decreasing trend in CA1 are also significantly correlated post HIIT/MICT. Thus, contrary to the similar increasing patterns of slow LFP rhythms, fast LFP rhythms, such as beta and gamma showed comparable decreasing tendencies in M1 or CA1 after HIIT/MICT. Moreover, HIIT and MICT showed similar potencies in decreasing the beta and gamma PSD. Summary of HIIT/MICT on slow and fast rhythm in M1 and CA1: MICT led to a more significant, more evident and prolonged increase of slow LFP rhythms, and a decrease of fast LFP rhythms, typically in CA1. Additionally, compared with fast rhythms, slow rhythm alterations are better biomarkers for CF post-HIIT/MICT.

### The effects of HIIT/MICT on GF in LFP

**Figure 5:**
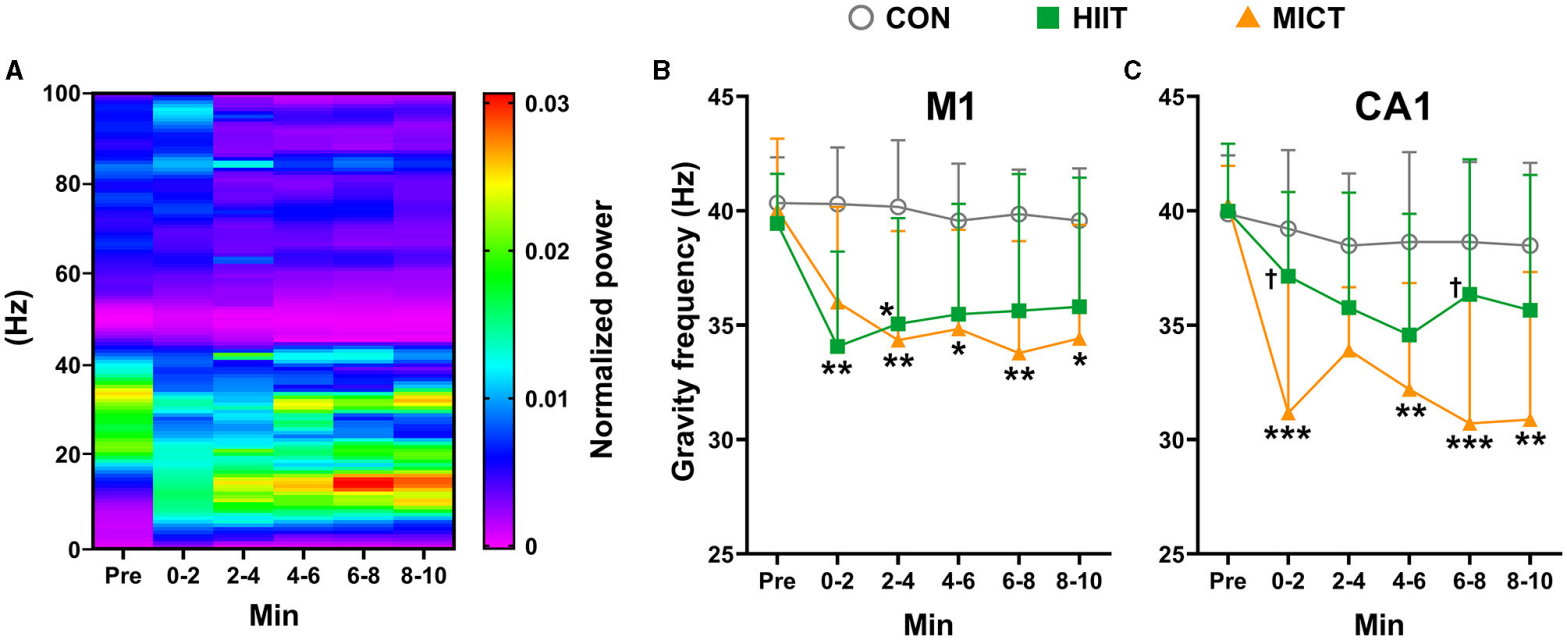
The GF of power spectrum alterations post-HIIT/MICT. **(A)** A represents LFP power spectrum alteration in CA1 post MICT in one mouse; **(B)** GF changes in M1 after HIIT/MICT; **(C)** GF changes in CA1 post HIIT/MICT. The data is represented by mean ± SD, *n* = 7–9. *p < 0.05, **p < 0.01 vs. control values; ^†^*p* < 0.05 vs. MICT values by Brown-Forsythe and Welch ANOVA followed by *Dunnett* multiple *post hoc* comparisons. ***p < 0.001 vs. control values.

Figure 5A depicts a represents LFP power spectrum alteration in CA1 in a mouse following MICT. In the present study, the pre-exercise value of GF in LFP is around 40 Hz in both M1 (39.94 ± 2.43 Hz, *n* = 25) and CA1 (40.05 ± 2.40 Hz, *n* = 25). Compared to the pre-exercise or control values, GF in both M1 and CA1 consistently decreased post HIIT/MICT, and greater decrease tendency in GF values were observed (Figures 5B, C), typically in CA1, following MICT (GF in CA1: 0–2 min, *p* < 0.05, 2–4, 4–6, 6–8 and 8–10 min, *p* < 0.05 vs. pre-exercise value by Brown-Forsythe and Welch ANOVA, Figures 5B, C). The results demonstrated a solid transition of the center of gravity components from fast to slow frequency band in both M1 and CA1, thus the cortical and subcortical dynamics were decreased and chaos level of multi-frequency components increased post-HIIT/MICT. The GF results indicate that CF induced by HIIT/MICT can partially be manifested by mental fatigue and a drop in attention level. Moreover, MICT fatigue is associated with the most significant GF reduction in CA1 (0–2 and 6–8 min, *p* < 0.05 vs. the GF values post-HIIT, Figure 5C). The finding parallels previous LFP coherence and PSD results. MICT fatigue is linked to more evident increased slow LFP rhythms, typically in CA1, and indicate more substantial and steadier suppression of functional integration between M1 and CA1.

### The effects of HIIT/MICT on neuronal firing rate

Neurons exchange and transmit information mostly through spikes, and neural spike trains in a variety of brain regions can be highly correlated. Therefore, besides LFP, we also checked the changes of neuronal firing characteristics post HIIT/MICT. In this study, a total of 418, 411, 396, 395, 397, and 393 M1 units, as well as 413, 392, 388, 386, 393, and 391 CA1 units, were identified during pre-immobile and at 0–2, 2–4, 4–6, 6–8, and 8–10 min respectively following 10 min of restriction on a stationary treadmill in nine control mice, and were used for analyses. We identified a total of 318, 293, 292, 285, 289, and 307 M1 units, and 315, 284, 273, 272, 256, and 281 CA1 units respectively during pre-exercise, 0–2, 2–4, 4–6, 6–8, and 8–10 min post-HIIT in seven mice, and these units were used for subsequent analyses. Similarly, a total of 406, 331, 276, 315, 232, and 203 M1 units, and 411, 344, 339, 314, 301, and 258 CA1 units were identified respectively during pre-exercise, 0–2, 2–4, 4–6, 6–8, and 8–10 min post-MICT in 9 mice, and these units were used for subsequent analyses. The units in pre-exercise phase in HIIT and MICT mice were recruited to obtain the overall average neuronal firing rate is 29.86 ± 10.65 Hz in M1 (1,142 units in 25 mice) and 25.84 ± 9.54 Hz in CA1 (1,139 units in 25 mice).

**Figure 6:**
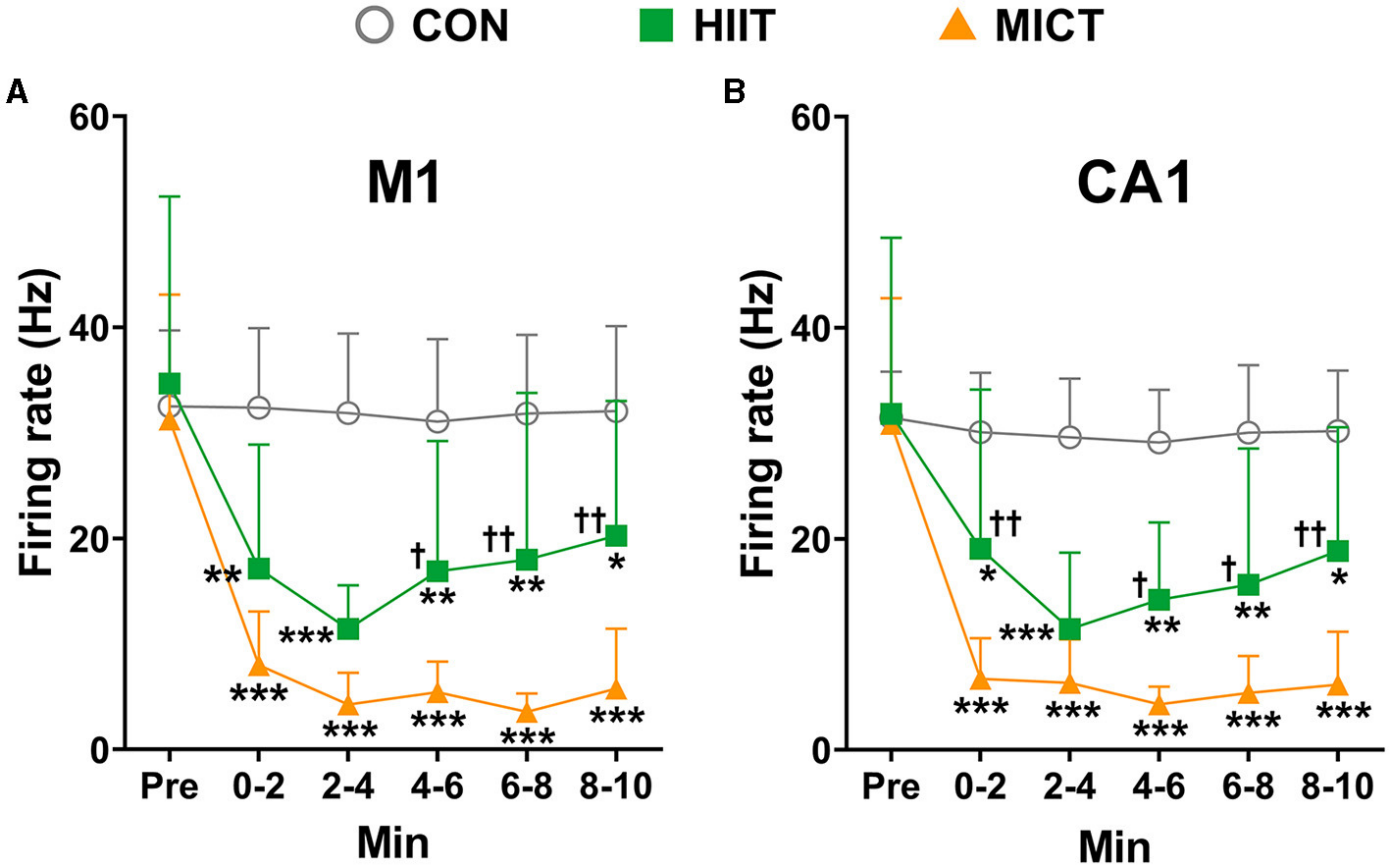
The general neuronal firing rate changes post-HIIT/MICT. **(A)** General firing rate changes in M1 after HIIT/MICT; **(B)** General firing rate changes in CA1 following HIIT/MICT. The data is represented by mean ± SD, *n* = 7–9. *p < 0.05, **p < 0.01, ***p < 0.001 vs. control values; ^†^*p* < 0.05, ^††^*p* < 0.01 vs. MICT values by Brown-Forsythe and Welch ANOVA followed by *Dunnett* multiple *post hoc* comparisons.

Compared to the pre-exercise or control values, the overall firing rates in both M1 (HIIT: 2–4 min, *p* < 0.05 vs. pre-exercise value by one-way ANOVA; MICT: 0–2, 2–4, 4–6, 6–8 and 8–10 min, *p* < 0.01 vs. pre-exercise value by Brown-Forsythe and Welch ANOVA, Figure 6A) and CA1 (MICT: 0–2, 2–4, 4–6, 6–8 and 8–10 min, *p* < 0.01 vs. pre-exercise value by Brown-Forsythe and Welch ANOVA, Figure 6B) consistently decreased post HIIT/MICT. Both M1 and CA1 firing rates exhibited a tendency toward recovery during the 10 min following HIIT. However, MICT-induced fatigue was associated with a more pronounced suppression of firing rates in M1 and CA1, particularly during the 4–10 min interval post-exercise, compared to HIIT-induced fatigue (Figures 6A, B). The results agree with the previous result that MICT fatigue is more profoundly linked to several LFP parameters.

### The effects of HIIT/MICT on cross-correlation of neuronal firing

**Figure 7:**
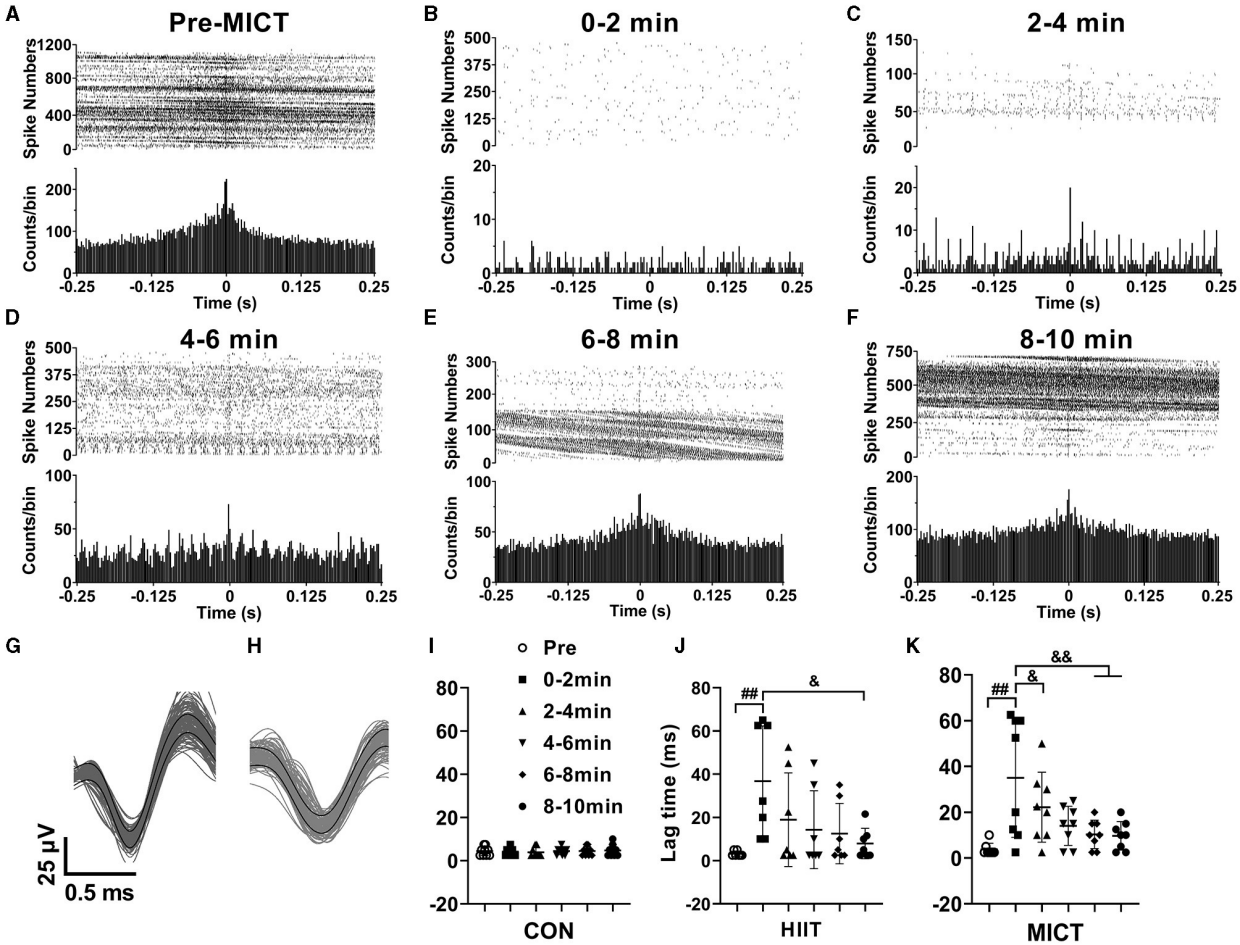
The cross-correlation alterations of spikes in M1 and CA1 post-HIIT/MICT. **(A–F)** Represent raster plots and cross-correlation histograms showing the alterations of spike synchrony pre- and post MICT in one mouse. **(G, H)** Typical waveforms of sorted neuronal firing in M1 and CA1 respectively; **(I–K)** Time lag changes of spikes in M1 and CA1 in control, HIIT and MICT mice respectively. The data is represented by mean ± SD, *n* = 7–9. ^##^*p* < 0.01 vs. pre-exercise value; ^&^*p* < 0.05, ^&&^*p* < 0.01 by one-way ANOVA followed by *Bonferroni* multiple comparisons.

Correlated neuronal firing closely linked to attention, stimulus discrimination, and motor function. Therefore, in this study, besides the LFP correlation, we also investigated the cross-correlation of neuronal firing in M1 and CA1. M1 and CA1 neurons fire simultaneously, and the high cross-correlation of neuronal firing was indicated by the evident single peak in the represent correlation histogram before MICT (CA1 spikes relative to M1 spikes, spike number = 18,207, Figure 7A). Neurons in M1 and CA1 tend to fire independently with least synchrony, as shown the represent flat the cross-correlogram (Salinas and Sejnowski, 2001) at 0–2 min post MICT (spike number = 280, Figure 7B). The peak in the correlation histograms 4–10 min post MICT (spike number = 626, 5,834, 8,742, 19,530 in Figures 7C–F respectively) gradually increases, indicating the synchrony of neuronal firings in M1 and CA1 progressively recovered, the overall spike densities gradually increase as shown in the raster plots (Figures 7B–F) compare to the pre-exercise value (Figure 7A).

Figures 7G, H illustrate representative waveforms of sorted neuronal firing activity in the M1 and CA1 regions, respectively. Furthermore, Figures 7I–K present the time lag variations of spikes in the M1 and CA1 regions of control, HIIT, and MICT mice, respectively. Before exercise, the meantime lag of CA1 neuron firing relative to M1 is 3.5 ± 2 ms (*n* = 16). The result indicates a temporal relationship between spikes from M1 and CA1, while normally CA1 neurons fire with 3.5 ms delay to M1 neurons. The time lag between M1 and CA1 spikes consistently increased post HIIT/MICT, and showed a clear tendency of recovery during 10 min post exercise. Thus, the synchrony of spike firing in M1 and CA1 largely recovered. However, there is no significant difference between the potency of HIIT and MICT on lag time increasing. Besides, it should be aware that the increasing degrees of synchrony not only shaped by signal propagating and synaptic processing along the neural network between M1 and CA1, but also influenced by common inputs.

### The effects of HIIT/MICT on burst firing properties

Neuronal burst firing is critical in specific coding and information transmission. For example, burst firing can enhance neural oscillations, increases neural output correlation and induce neural plasticity (Chan et al., 2016). Atypical burst firing is implicated in a series of mental dysfunction and neural neurological disorders, including anxiety, depression, and epilepsy (Shao et al., 2021). Therefore, in the present study, we also assessed alternation of burst firing features post HIIT/MICT. Applying the criteria and identify approaches mentioned in the method, the burst units were identified in pre and every 2 min post HIIT/MICT. In this study, a total of 62, 59, 57, 54, 56 and 53 M1 burst units, and a total of 64, 61, 66, 52, 53 and 58 CA1 burst units were identified during pre-immobile and at 0–2, 2–4, 4–6, 6–8, and 8–10 min respectively following 10 min of restriction on a stationary treadmill in 9 control mice, and were used for analyses. Moreover, we identified a total of 41, 29, 28, 32, 34, and 35 M1 burst units, and 43, 31, 33, 34, 36, and 38 CA1 burst units during pre-exercise, 0–2, 2–4, 4–6, 6–8, and 8–10 min post-HIIT in seven mice, and used them for analysis. Similarly, in nine MICT mice, a total of 53, 42, 44, 46, 50, and 53 M1 burst units, and 62, 48, 50, 53, 56, and 59 CA1 burst units respectively were identified during pre-exercise, 0–2, 2–4, 4–6, 6–8, and 8–10 min post-MICT and used for subsequent analyses.

**Figure 8:**
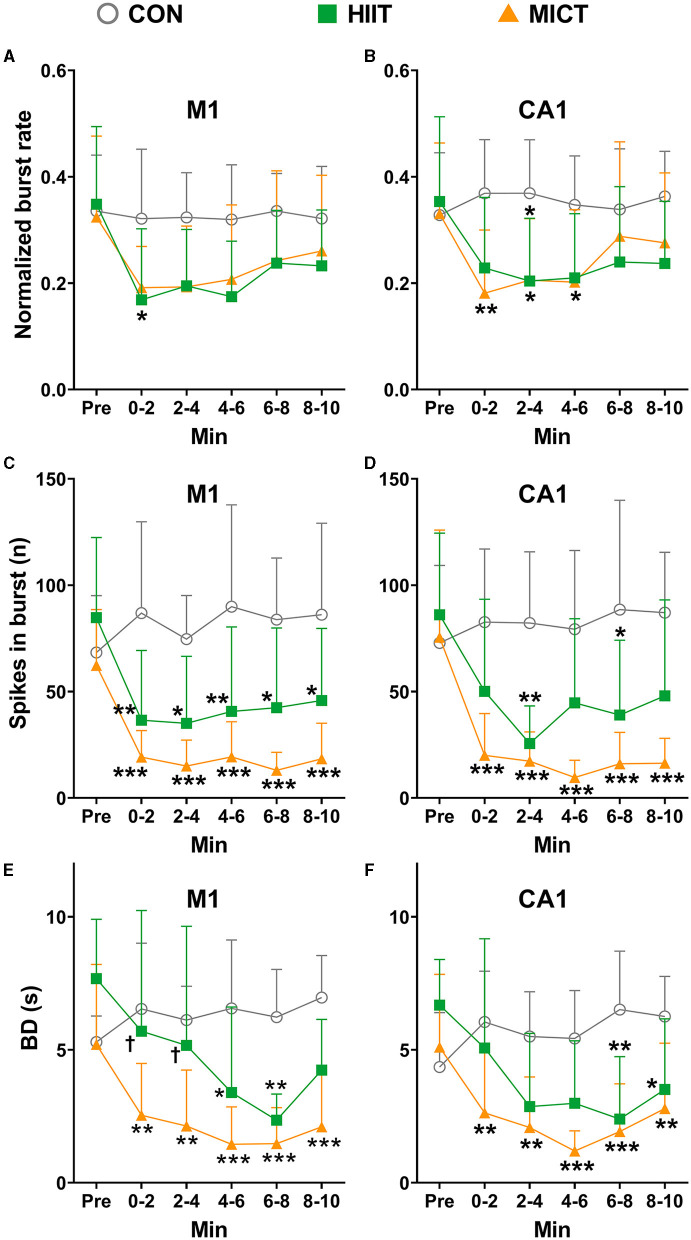
The normalized burst firing rate, spikes in burst and BD changes after HIIT/MICT. **(A, B)** Normalized burst firing rate (relative to total firing rate) changes in M1 and CA1 after HIIT/MICT; **(C, D)** The alternation of spikes in burst in M1 and CA1 following HIIT/MICT; **(E, F)** BD changes in M1 and CA1 after HIIT/MICT. The data is represented by mean ± SD, *n* = 7–9. *p < 0.05, **p < 0.01; ***p < 0.001 vs. control values; ^†^*p* < 0.05 vs. MICT values by Brown-Forsythe and Welch ANOVA followed by *Dunnett* multiple *post hoc* comparisons.

Normalized to the overall neuronal units, the ratio of M1 burst units are 0.13 and 0.11, and the ratio of CA1 burst units are 0.14 and 0.13 in seven mice during pre-exercise and 8–10 min post HIIT respectively; while the ratio of M1 burst units are 0.13 and 0.26, and the ratio of CA1 burst units are 0.15 and 0.23 in nine mice during pre-exercise and 8–10 min post HIIT respectively. The normalized burst rates relative to overall all firing frequencies were calculated (Figure 8A). Before exercise, the normalized mean burst rates in M1 and CA1 are 0.34 ± 0.13 (156 burst units in 25 mice) and 0.34 ± 0.14 (169 burst units in 25 mice) respectively. Concomitantly, the mean spike number in burst is 71.81 ± 30.19 in M1 and 78.16 ± 41.71 in CA1.

Consistent with the changes observed in overall firing frequency, the burst features in both M1 and CA1 also exhibited a consistent inhibition post-HIIT/MICT, as compared to pre-exercise or control values, as reflected by the reduced burst rates (Figures 8A, B), number of spikes in burst (Figures 8C, D) and burst duration (Figures 8E, F). Significant decreases in the number of spikes in burst were observed in MICT mice during 0–2, 2–4, 4–6, 6–8 and 8–10 min post-exercise (*p* < 0.05 for M1 vs. pre-exercise values by Brown-Forsythe and Welch ANOVA and *p* < 0.01 for CA1 vs. pre-exercise values by one-way ANOVA, Figures 8C, D).

Before exercise, the mean BD in M1 and CA1 are 6.06 ± 2.07 s (*n* = 25) and 5.38 ± 2.16 s (*n* = 25) respectively. The BD in both M1 and CA1 (MICT: 4–6 min, *p* < 0.05 vs. pre-exercise value by Brown-Forsythe and Welch ANOVA) reduced after HIIT/MICT. Consistent with the alterations in normalized burst rate, spikes in burst, the decrease in BD was steadier after MICT compared with the counterpart values after HIIT (Figures 8E, F). Combined with the burst ratio data, the overall alterations in burst features indicated that a large portion of burst units transited from long burst with more dense spikes to short burst with fewer spikes, and this conversion is steadier in MICT mice. Thus, compared with HIIT, MICT appears to be similarly effective in altering burst characteristics.

## Discussion

The mechanism for CF during strenuous exercise, such as HIIT and MICT remains elusive, with limited research in this field. Recent studies suggest that the inception of CF lies within the brain (Tornero-Aguilera et al., 2022). While diminished executive processing in the motor cortex has been implicated, it is plausible that circuit-level changes across motor cortex and hippocampal regions may contribute to the depression, feeling of fatigue, and cognitive decline observed in exercise fatigue. Here, we employed an adapted mouse model of HIIT/MICT and concurrent extracellular recordings in the M1 and CA1 regions to systematically examine alterations in LFP and spike activities within 10 min post-exercise. Our findings indicate that both HIIT and MICT consistently reduced LFP coherence in M1 and CA1 across slow (delta, theta, alpha) and fast (beta, gamma) frequency bands. Furthermore, we observed consistent increases in delta, theta, and alpha power, along with reductions in beta and gamma power in both regions. Notably, GF steadily decreased in both M1 and CA1 following HIIT/MICT. The altered LFP and spike activities in the M1 and CA1 regions following HIIT/MICT may reflect compensatory neural adaptations, analogous to those reported in adult cognitive fatigue studies (Wang et al., 2016; Babu Henry Samuel et al., 2019), and may serve a protective role during maximal or endurance exercise (Noakes, 2012).

EEG/LFP is associated with different behavioral and cognitive processes with certain brain regions and oscillatory dynamics. For instance, cortical delta rhythm (1–4 Hz) is prevalent during drowsiness and sleep (Davis et al., 2011). Hippocampal theta rhythm is associated with episodic memory and navigation (Buzsáki and Moser, 2013). However, fatigue is characterized by an increase in cortical delta and theta, accompanied by a decrease in beta activity (Lal and Craig, 2001; Zinn et al., 2018). Furthermore, chronic pain is associated with elevated theta and alpha power at rest, as well as a reduced amplitude of evoked potentials following sensory stimulation and cognitive tasks, indicating its complex neurophysiological underpinnings (Pinheiro et al., 2016). Alpha rhythm (8–14 Hz), being the dominant pattern in the awake brain, is associated with sensory processing and attention (Zhou et al., 2021), likely modulating the excitability level of the brain's internal state through functional inhibition (Jensen and Mazaheri, 2010). Alpha oscillations have been shown to exhibit a negative correlation with level of alertness, attention and fatigue (Gharagozlou et al., 2015). Cortical beta rhythm (15–30 Hz) reflects processes related to working memory and somatosensory decision-making (Haegens et al., 2011, 2017; Lundqvist et al., 2018). Gamma rhythm (30–90 Hz) is associated with sensory processing, cognition, memory, and attention across various brain regions (Lundqvist et al., 2018). The somatosensory regions showed a stimulus-evoked response, reflected in a power increase in the beta (Haegens et al., 2011, 2017) and gamma range (Haegens et al., 2011). The fluctuations observed in delta, theta, alpha, beta, and gamma rhythms in our study likely reflect changes in emotional, memorial, cognitive, and sensory process in HIIT/MICT induced fatigue.

The temporal correlation in LFP between two brain regions is typically quantified by coherence, reflecting the phase consistency of neural excitability across sites (Srinath and Ray, 2014). Our findings of reduced LFP coherence post-HIIT/MICT indicate that both exercise modalities impact local neural circuit activities and functional integration between M1 and CA1. Mental fatigue, a psychobiological state resulting from prolonged exertion, impairs cognitive abilities and exercise performance (Meeusen et al., 2021). This state hinders psychomotor vigilance (Angius et al., 2022), diminishes exercise pleasure, and negatively impacts implicit attitudes toward future exercise (Pessoa et al., 2022). The reduction in GF strongly correlates with mental fatigue (Li H. et al., 2019), while PSE (chaos level) positively correlated with attention level (Frohlich et al., 2021). Our GF analysis revealed a shift in the spectral center of gravity from faster to slower frequency bands, accompanied by an elevated PSE after HIIT/MICT, suggesting mental fatigue and decreased attention. These results align with the notion that fatigue can stem from diverse origins, not solely physiological factors (Tornero-Aguilera et al., 2022). Therefore, the alterations in LFP oscillations, LFP coherence, and GF collectively depict a dynamic, distributed neuronal network in the M1 and CA1, contributing to the diverse fatigue manifestations induced by HIIT/MICT.

This study reveals that MICT, in contrast to HIIT, exhibits greater efficacy in disrupting temporal correlations of LFP activities between M1 and CA1, augmenting slow LFP rhythms, inhibiting fast LFP oscillations, and reducing GF in CA1. Notably, alterations in slow rhythms, compared to fast rhythms, emerge as superior biomarkers for cognitive function following HIIT/MICT. Our findings align with prior research indicating the hippocampus's involvement in theta wave generation, which facilitates motor performance (Huang et al., 2005; Bohbot et al., 2017). Given the substantial similarities in EEG/LFP activity between human and rodent hippocampi (Bohbot et al., 2017), the current findings hold significant implications for exploring LFP and CF alterations in humans. Exercise fatigue exerts multifaceted impacts on psychological and physiological functions (Tornero-Aguilera et al., 2022). CF, in particular, deteriorates cognitive functions and mood. The differential impact of HIIT and MICT on LFP activity may elucidate the more pronounced CF induced by MICT compared to HIIT. Our findings align with previous studies indicating that MICT elicits more CF components than HIIT in human (Burnley and Jones, 2018; Tornero-Aguilera et al., 2022). The synchronized and significant post-exercise alterations in LFP activity in M1 and CA1 observed in our study are consistent with CA1's involvement in motor alterations associated with fatigue in forced swimming rats (Chen et al., 2009).

The hippocampus plays a crucial role in consolidating motor sequence memory (Albouy et al., 2008; Jacobacci et al., 2020), and is functionally connected to the sensorimotor cortex during volitional movements (Burman, 2019). In this study, both HIIT and MICT influenced the cross-correlation of neuronal firing between the M1 and CA1, with MICT exhibiting a more potent effect. The effects of HIIT/MICT on neuronal firing are characterized by delayed time lags, reduced overall firing rates, burst rates and spikes in burst, as well as decreased BD. Notably, MICT is more effective in decreasing general firing rates in both the M1 and CA1. While common inputs or synaptic interactions can influence neuronal cross-correlation, these correlations still offer crucial insights into neural network functional architecture (Salinas and Sejnowski, 2001; Cohen and Kohn, 2011). Our cross-correlation data aligns with previous LFP coherence findings, indicating a consistent decrease in neural activity synchronization between M1 and CA1, and reflecting a downregulation of functional integration across these regions.

Neuronal burst firing plays a pivotal role in specific coding and information transmission. It can enhance neural oscillations, increase neural output correlation, and induce neural plasticity (Chan et al., 2016). Aberrant burst firing patterns have been implicated in a range of mental dysfunctions and neurological disorders, such as anxiety, depression, and epilepsy (Shao et al., 2021). In the current study, we evaluated the alterations in burst firing characteristics following HIIT and MICT. Notably, MICT showed greater effectiveness in modifying burst firing rate, spikes in burst and BD in M1 and CA1. Further, all burst parameters except BD displayed a significant recovery trend within 10 min post-exercise. These results indicate that both HIIT and MICT can impact neural information coding and transmission by modulating burst firing in distinct brain regions, potentially contributing to the negative mental perceptions associated with these exercise modalities.

Rodent studies have implicated the involvement of neurotransmitters such as dopamine, serotonin, and noradrenaline in exercise-induced CF across multiple brain regions (Meeusen and Roelands, 2018; Meeusen et al., 2021; Tornero-Aguilera et al., 2022). However, the results of animal and human studies are not fully consistent in the relationship between dopamine, serotonin, and exercise fatigue (Cordeiro et al., 2017; Yan et al., 2022). Thus, the precise mechanism underlying the occurrence of CF still remains elusive (Meeusen and Roelands, 2018). Our research also has limitations. Specifically, the modifications of neural network and neuronal firing observed in this study were obtained from strenuous mouse exercise models, potentially limiting the direct extrapolation to human brain neural network changes associated with CF. Nevertheless, compelling evidence suggests functional similarities between human (Kunz et al., 2024) and rodent (Khodagholy et al., 2017; Nitzan et al., 2020) brains, particularly in the connectivity between the hippocampus and cortex, mediated by the entorhinal region and default mode network, as reflected in comparable EEG/LFP activity (Bohbot et al., 2017). Hence, the alterations in neural network connectivity between M1 and CA1 regions identified in this study offer a valuable perspective for interpreting exercise-induced CF in humans. Furthermore, our findings align with recent research demonstrating ultrastructural changes in asymmetric synapses within the rat striatum along cortical-striatal pathways, associated with repeated exercise-induced fatigue (Wang et al., 2019).

## Conclusions

This study provides novel evidence that both HIIT and MICT significantly modulate neural activities in M1 and CA1 regions. The alterations observed in LFP and neuronal firing patterns could potentially serve as biomarkers for motor disturbances and fatigue-related brain signals. Our findings indicate that MICT demonstrates a stronger correlation with changes in LFP and neuronal firing parameters. Additionally, MICT-induced fatigue seems to be accompanied by a more pronounced disturbance in the functional integration between M1 and CA1. These observations align with previous studies suggesting that MICT leads to higher levels of CF compared to HIIT (Burnley and Jones, 2018; Tornero-Aguilera et al., 2022). Our study sheds light on the neurophysiological mechanisms underlying exercise-induced CF within cortical and subcortical networks. Notably, our findings reveal compensatory and self-protective brain mechanisms activated during exhaustive exercise. These insights have the potential to guide exercise programming to optimize performance while minimizing excessive fatigue. Additionally, our research may contribute to the development of targeted therapeutic interventions for clinical conditions associated with exercise-induced fatigue.

## Data Availability

The original contributions presented in the study are included in the article, further inquiries can be directed to the corresponding author.

## References

[B1] Ahmadi N. Constandinou T. G. Bouganis C. S. (2021). Inferring entire spiking activity from local field potentials. Sci. Rep. 11:19045. 10.1038/s41598-021-98021-9 34561480 PMC8463692

[B2] Albouy G. Sterpenich V. Balteau E. Vandewalle G. Desseilles M. Dang-Vu T.. (2008). Both the hippocampus and striatum are involved in consolidation of motor sequence memory. Neuron 58, 261–272. 10.1016/j.neuron.2008.02.008 18439410

[B3] Anders J. P. V. Kraemer W. J. Newton R. U. Post E. M. Caldwell L. K. Beeler M. K.. (2021). Acute effects of high-intensity resistance exercise on cognitive function. J. Sports Sci. Med. 20, 391–397. 10.52082/jssm.2021.391 34267577 PMC8256515

[B4] Angius L. Merlini M. Hopker J. Bianchi M. Fois F. Piras F.. (2022). Physical and mental fatigue reduce psychomotor vigilance in professional football players. Int. J. Sports Physiol. Perform. 17, 1391–1398. 10.1123/ijspp.2021-0387 35477898

[B5] Babu Henry Samuel I. Wang C. Burke S. E. Kluger B. Ding M. (2019). Compensatory neural responses to cognitive fatigue in young and older adults. Front. Neural. Circuits 13:12. 10.3389/fncir.2019.00012 30853901 PMC6396034

[B6] Baker E. J. Gleeson T. T. (1999). The effects of intensity on the energetics of brief locomotor activity. J. Exp. Biol. 202, 3081–3087. 10.1242/jeb.202.22.3081 10539956

[B7] Bohbot V. D. Copara M. S. Gotman J. Ekstrom A. D. (2017). Low-frequency theta oscillations in the human hippocampus during real-world and virtual navigation. Nat. Commun. 8:14415. 10.1038/ncomms14415 28195129 PMC5316881

[B8] Burman D. D. (2019). Hippocampal connectivity with sensorimotor cortex during volitional finger movements: Laterality and relationship to motor learning. PLoS ONE 14:e0222064. 10.1371/journal.pone.0222064 31536543 PMC6752792

[B9] Burnley M. Jones A. M. (2018). Power-duration relationship: physiology, fatigue, and the limits of human performance. Eur. J. Sport Sci. 18, 1–12. 10.1080/17461391.2016.1249524 27806677

[B10] Buzsáki G. Moser E. I. (2013). Memory, navigation and theta rhythm in the hippocampal-entorhinal system. Nat. Neurosci. 16, 130–138. 10.1038/nn.3304 23354386 PMC4079500

[B11] Carroll T. J. Taylor J. L. Gandevia S. C. (2017). Recovery of central and peripheral neuromuscular fatigue after exercise. J. Appl. Physiol. 122, 1068–1076. 10.1152/japplphysiol.00775.2016 27932676

[B12] Chan H. K. Yang D. P. Zhou C. Nowotny T. (2016). Burst firing enhances neural output correlation. Front. Comput. Neurosci. 10:42. 10.3389/fncom.2016.00042 27242499 PMC4860405

[B13] Chen J. R. Wang T. J. Huang H. Y. Chen L. J. Huang Y. S. Wang Y. J.. (2009). Fatigue reversibly reduced cortical and hippocampal dendritic spines concurrent with compromise of motor endurance and spatial memory. Neuroscience 161, 1104–1113. 10.1016/j.neuroscience.2009.04.022 19376203

[B14] Cohen M. R. Kohn A. (2011). Measuring and interpreting neuronal correlations. Nat. Neurosci. 14, 811–819. 10.1038/nn.2842 21709677 PMC3586814

[B15] Cordeiro L. M. S. Rabelo P. C. R. Moraes M. M. Teixeira-Coelho F. Coimbra C. C. Wanner S. P.. (2017). Physical exercise-induced fatigue: the role of serotonergic and dopaminergic systems. Braz. J. Med. Biol. Res. 50:e6432. 10.1590/1414-431x20176432 29069229 PMC5649871

[B16] Crouch B. Sommerlade L. Veselcic P. Riedel G. Schelter B. Platt B.. (2018). Detection of time-, frequency- and direction-resolved communication within brain networks. Sci. Rep. 8:1825. 10.1038/s41598-018-19707-1 29379037 PMC5788985

[B17] Davis C. J. Clinton J. M. Jewett K. A. Zielinski M. R. Krueger J. M. (2011). Delta wave power: an independent sleep phenotype or epiphenomenon? J. Clin. Sleep Med. 7, S16–S18. 10.5664/JCSM.1346 22003323 PMC3190419

[B18] De Sousa Fernandes M. S. Ordônio T. F. Santos G. C. J. Santos L. E. R. Calazans C. T. Gomes D. A.. (2020). Effects of physical exercise on neuroplasticity and brain function: a systematic review in human and animal studies. Neural. Plast. 2020:8856621. 10.1155/2020/8856621 33414823 PMC7752270

[B19] Dougherty J. P. Springer D. A. Gershengorn M. C. (2016). The treadmill fatigue test: a simple, high-throughput assay of fatigue-like behavior for the mouse. J. Vis. Exp. 111:54052. 10.3791/54052-v 27286034 PMC4927751

[B20] Einevoll G. T. Kayser C. Logothetis N. K. Panzeri S. (2013). Modelling and analysis of local field potentials for studying the function of cortical circuits. Nat. Rev. Neurosci. 14, 770–785. 10.1038/nrn3599 24135696

[B21] Fernández-Seara M. A. Aznárez-Sanado M. Mengual E. Loayza F. R. Pastor M. A. (2009). Continuous performance of a novel motor sequence leads to highly correlated striatal and hippocampal perfusion increases. Neuroimage 47, 1797–1808. 10.1016/j.neuroimage.2009.05.061 19481611

[B22] Frohlich J. Toker D. Monti M. M. (2021). Consciousness among delta waves: a paradox? Brain 144, 2257–2277. 10.1093/brain/awab095 33693596

[B23] Gharagozlou F. Nasl Saraji G. Mazloumi A. Nahvi A. Motie Nasrabadi A. Rahimi Foroushani A.. (2015). Detecting driver mental fatigue based on EEG alpha power changes during simulated driving. Iran. J. Public Health 44, 1693–1700. 26811821 PMC4724743

[B24] Gorham L. S. Jernigan T. Hudziak J. Barch D. M. (2019). Involvement in sports, hippocampal volume, and depressive symptoms in children. Biol. Psychiatry Cogn. Neurosci. Neuroimaging 4, 484–492. 10.1016/j.bpsc.2019.01.011 30905689 PMC6500760

[B25] Haegens S. Nácher V. Hernández A. Luna R. Jensen O. Romo R.. (2011). Beta oscillations in the monkey sensorimotor network reflect somatosensory decision making. Proc. Natl. Acad. Sci. U S A 108, 10708–10713. 10.1073/pnas.1107297108 21670296 PMC3127887

[B26] Haegens S. Vergara J. Rossi-Pool R. Lemus L. Romo R. (2017). Beta oscillations reflect supramodal information during perceptual judgment. Proc. Natl. Acad. Sci. U S A 114, 13810–13815. 10.1073/pnas.1714633115 29229820 PMC5748206

[B27] Herreras O. (2016). Local field potentials: myths and misunderstandings. Front. Neural. Circuits 10:101. 10.3389/fncir.2016.00101 28018180 PMC5156830

[B28] Huang Y. Z. Edwards M. J. Rounis E. Bhatia K. P. Rothwell J. C. (2005). Theta burst stimulation of the human motor cortex. Neuron 45, 201–206. 10.1016/j.neuron.2004.12.033 15664172

[B29] Ibarra-Lecue I. Haegens S. Harris A. Z. (2022). Breaking down a rhythm: dissecting the mechanisms underlying task-related neural oscillations. Front. Neural. Circuits 16:846905. 10.3389/fncir.2022.846905 35310550 PMC8931663

[B30] Jacobacci F. Armony J. L. Yeffal A. Lerner G. Amaro E. Jr. Jovicich J.. (2020). Rapid hippocampal plasticity supports motor sequence learning. Proc. Natl. Acad. Sci. U S A 117, 23898–23903. 10.1073/pnas.2009576117 32900965 PMC7519327

[B31] Jensen O. Mazaheri A. (2010). Shaping functional architecture by oscillatory alpha activity: gating by inhibition. Front. Hum. Neurosci. 4:186. 10.3389/fnhum.2010.00186 21119777 PMC2990626

[B32] Jonak C. R. Lovelace J. W. Ethell I. M. Razak K. A. Binder D. K. (2018). Reusable multielectrode array technique for electroencephalography in awake freely moving mice. Front. Integr. Neurosci. 12:53. 10.3389/fnint.2018.00053 30416434 PMC6213968

[B33] Khodagholy D. Gelinas J. N. Buzsáki G. (2017). Learning-enhanced coupling between ripple oscillations in association cortices and hippocampus. Science 358, 369–372. 10.1126/science.aan6203 29051381 PMC5872145

[B34] Kunz L. Staresina B. P. Reinacher P. C. Brandt A. Guth T. A. Schulze-Bonhage A.. (2024). Ripple-locked coactivity of stimulus-specific neurons and human associative memory. Nat. Neurosci. 27, 587–599. 10.1038/s41593-023-01550-x 38366143 PMC10917673

[B35] Lal S. K. Craig A. (2001). A critical review of the psychophysiology of driver fatigue. Biol. Psychol. 55, 173–194. 10.1016/S0301-0511(00)00085-5 11240213

[B36] Leavitt V. M. DeLuca J. (2010). Central fatigue: issues related to cognition, mood and behavior, and psychiatric diagnoses. PM R 2, 332–337. 10.1016/j.pmrj.2010.03.027 20656614

[B37] Lee S. M. Kim Y. H. Kim Y. R. Lee B. R. Shin S. Kim J. Y.. (2022). Anti-fatigue potential of *Pinus koraiensis* leaf extract in an acute exercise-treated mouse model. Biomed. Pharmacother. 153:113501. 10.1016/j.biopha.2022.113501 36076511

[B38] Legendy C. R. Salcman M. (1985). Bursts and recurrences of bursts in the spike trains of spontaneously active striate cortex neurons. J. Neurophysiol. 53, 926–939. 10.1152/jn.1985.53.4.926 3998798

[B39] Li B. Liang F. Ding X. Yan Q. Zhao Y. Zhang X.. (2019). Interval and continuous exercise overcome memory deficits related to beta-amyloid accumulation through modulating mitochondrial dynamics. Behav. Brain Res. 376:112171. 10.1016/j.bbr.2019.112171 31445975

[B40] Li H. Wang D. Chen J. Luo X. Li J. Xing X.. (2019). Pre-service fatigue screening for construction workers through wearable EEG-based signal spectral analysis. Autom. Constr. 106:102851. 10.1016/j.autcon.2019.102851

[B41] Lundqvist M. Herman P. Warden M. R. Brincat S. L. Miller E. K. (2018). Gamma and beta bursts during working memory readout suggest roles in its volitional control. Nat. Commun. 9:394. 10.1038/s41467-017-02791-8 29374153 PMC5785952

[B42] Meeusen R. Roelands B. (2018). Fatigue: is it all neurochemistry? Eur. J. Sport Sci. 18, 37–46. 10.1080/17461391.2017.1296890 28317427

[B43] Meeusen R. Van Cutsem J. Roelands B. (2021). Endurance exercise-induced and mental fatigue and the brain. Exp. Physiol. 106, 2294–2298. 10.1113/EP088186 32176398

[B44] Moore R. D. Romine M. W. O'connor P, J. Tomporowski P.D. (2012). The influence of exercise-induced fatigue on cognitive function. J. Sports Sci. 30, 841–850. 10.1080/02640414.2012.675083 22494399

[B45] Nitzan N. Mckenzie S. Beed P. English D. F. Oldani S. Tukker J. J.. (2020). Propagation of hippocampal ripples to the neocortex by way of a subiculum-retrosplenial pathway. Nat. Commun. 11:1947. 10.1038/s41467-020-15787-8 32327634 PMC7181800

[B46] Noakes T. D. (2012). Fatigue is a brain-derived emotion that regulates the exercise behavior to ensure the protection of whole body homeostasis. Front. Physiol. 3:82. 10.3389/fphys.2012.00082 22514538 PMC3323922

[B47] Orellana V. D. Donoghue J. P. Vargas-Irwin C. E. (2024). Low frequency independent components: internal neuromarkers linking cortical LFPs to behavior. iScience 27:108310. 10.1016/j.isci.2023.108310 38303697 PMC10831875

[B48] Ouyang W. Yan Q. Zhang Y. Fan Z. (2017). Moderate injury in motor-sensory cortex causes behavioral deficits accompanied by electrophysiological changes in mice adulthood. PLoS ONE 12:e0171976. 10.1371/journal.pone.0171976 28196142 PMC5308857

[B49] Pessoa F. A. Pereira L. C. De Oliveira Araújo A. Oliveira G. T. A. Pereira D. C. Elsangedy H. M. (2022). Mental fatigue prior to aerobic exercise reduces exercise pleasure and negatively affects implicit attitudes toward future exercise. Percept. Mot. Skills 129, 816–832. 10.1177/00315125221091158 35435053

[B50] Pinheiro E. S. De Queirós F. C. Montoya P. Santos C. L. Do Nascimento M. A. Ito C. H.. (2016). Electroencephalographic patterns in chronic pain: a systematic review of the literature. PLoS ONE 11:e0149085. 10.1371/journal.pone.0149085 26914356 PMC4767709

[B51] Prathap S. Nagel B. J. Herting M. M. (2021). Understanding the role of aerobic fitness, spatial learning, and hippocampal subfields in adolescent males. Sci. Rep. 11:9311. 10.1038/s41598-021-88452-9 33927247 PMC8084987

[B52] Price R. B. Duman R. (2020). Neuroplasticity in cognitive and psychological mechanisms of depression: an integrative model. Mol. Psychiatry 25, 530–543. 10.1038/s41380-019-0615-x 31801966 PMC7047599

[B53] Rodriguez-Ayllon M. Cadenas-Sánchez C. Estévez-López F. Muñoz N. E. Mora-Gonzalez J. Migueles J. H.. (2019). Role of physical activity and sedentary behavior in the mental health of preschoolers, children and adolescents: a systematic review and meta-analysis. Sports Med. 49, 1383–1410. 10.1007/s40279-019-01099-5 30993594

[B54] Salinas E. Sejnowski T. J. (2001). Correlated neuronal activity and the flow of neural information. Nat. Rev. Neurosci. 2, 539–550. 10.1038/35086012 11483997 PMC2868968

[B55] Seldeen K. L. Redae Y. Z. Thiyagarajan R. Berman R. N. Leiker M. M. Troen B. R.. (2019). Short session high intensity interval training and treadmill assessment in aged mice. J. Vis. Exp. 144:10.3791/59138. 10.3791/59138-v 30774134 PMC9897322

[B56] Shao J. Liu Y. Gao D. Tu J. Yang F. (2021). Neural burst firing and its roles in mental and neurological disorders. Front. Cell Neurosci. 15:741292. 10.3389/fncel.2021.741292 34646123 PMC8502892

[B57] Singh B. Wang Z. Constantinidis C. (2023). Neuronal selectivity for stimulus information determines prefrontal LFP gamma power regardless of task execution. Commun. Biol. 6:505. 10.1038/s42003-023-04855-6 37169826 PMC10175284

[B58] Smallwood J. Bernhardt B. C. Leech R. Bzdok D. Jefferies E. Margulies D. S.. (2021). The default mode network in cognition: a topographical perspective. Nat. Rev. Neurosci. 22, 503–513. 10.1038/s41583-021-00474-4 34226715

[B59] Srinath R. Ray S. (2014). Effect of amplitude correlations on coherence in the local field potential. J. Neurophysiol. 112, 741–751. 10.1152/jn.00851.2013 24790174 PMC4122739

[B60] Sujkowski A. Hong L. Wessells R. J. Todi S. V. (2022). The protective role of exercise against age-related neurodegeneration. Ageing Res. Rev. 74:101543. 10.1016/j.arr.2021.101543 34923167 PMC8761166

[B61] Taylor J. M. Montgomery M. H. Gregory E. J. Berman N. E. (2015). Exercise preconditioning improves traumatic brain injury outcomes. Brain Res. 1622, 414–429. 10.1016/j.brainres.2015.07.009 26165153 PMC4562892

[B62] Tornero-Aguilera J. F. Jimenez-Morcillo J. Rubio-Zarapuz A. Clemente-Suárez V. J. (2022). Central and peripheral fatigue in physical exercise explained: a narrative review. Int. J. Environ. Res. Public Health 19:3909. 10.3390/ijerph19073909 35409591 PMC8997532

[B63] Voss M. W. Prakash R. S. Erickson K. I. Basak C. Chaddock L. Kim J. S.. (2010). Plasticity of brain networks in a randomized intervention trial of exercise training in older adults. Front. Aging Neurosci. 2:32. 10.3389/fnagi.2010.00032 20890449 PMC2947936

[B64] Wang C. Trongnetrpunya A. Samuel I. B. Ding M. Kluger B. M. (2016). Compensatory neural activity in response to cognitive fatigue. J. Neurosci. 36, 3919–3924. 10.1523/JNEUROSCI.3652-15.2016 27053200 PMC4821906

[B65] Wang Z. Hou L. Wang D. (2019). Effects of exercise-induced fatigue on the morphology of asymmetric synapse and synaptic protein levels in rat striatum. Neurochem. Int. 129:104476. 10.1016/j.neuint.2019.104476 31145967

[B66] Weavil J. C. Amann M. (2019). Neuromuscular fatigue during whole body exercise. Curr. Opin. Physiol. 10, 128–136. 10.1016/j.cophys.2019.05.008 32818161 PMC7430763

[B67] Weng T. B. Pierce G. L. Darling W. G. Falk D. Magnotta V. A. Voss M. W.. (2017). The acute effects of aerobic exercise on the functional connectivity of human brain networks. Brain Plast. 2, 171–190. 10.3233/BPL-160039 29765855 PMC5928541

[B68] Xu M. Liang R. Li Y. Wang J. (2017). Anti-fatigue effects of dietary nucleotides in mice. Food. Nutr. Res. 61:1334485. 10.1080/16546628.2017.1334485 28659748 PMC5475326

[B69] Yan K. Gao H. Liu X. Zhao Z. Gao B. Zhang L.. (2022). Establishment and identification of an animal model of long-term exercise-induced fatigue. Front. Endocrinol. 13:915937. 10.3389/fendo.2022.915937 36093084 PMC9459130

[B70] Zheng C. Colgin L. L. (2015). Beta and gamma rhythms go with the flow. Neuron 85, 236–237. 10.1016/j.neuron.2014.12.067 25611505 PMC4467988

[B71] Zhou Y. J. Iemi L. Schoffelen J. M. De Lange F. P. and Haegens S. (2021). Alpha oscillations shape sensory representation and perceptual sensitivity. J. Neurosci. 41, 9581–9592. 10.1523/JNEUROSCI.1114-21.2021 34593605 PMC8612475

[B72] Zinn M. A. Zinn M. L. Valencia I. Jason L. A. Montoya J. G. (2018). Cortical hypoactivation during resting EEG suggests central nervous system pathology in patients with chronic fatigue syndrome. Biol. Psychol. 136, 87–99. 10.1016/j.biopsycho.2018.05.016 29802861 PMC6064389

