# Correction: Comparison of alterations in local field potentials and neuronal firing in mouse M1 and CA1 associated with central fatigue induced by high-intensity interval training and moderate-intensity continuous training

**DOI:** 10.3389/fnins.2026.1976840

**Published:** 2026-09-08

**Authors:** Yuncheng Liu, Weiyi Lao, Haojie Mao, Yaoyao Zhong, Jihui Wang, Wei Ouyang

**Affiliations:** College of Physical Education and Health Sciences, Zhejiang Normal University, Jinhua, China

**Keywords:** exhausting exercise, primary motor cortex, hippocampal CA1, local field potentials, neuronal firing

In the **Acknowledgments**, the authors erroneously included a comment thanking a fourth-year medical student at UPenn Nursing School, Bachelor of Science in Nursing Program, Philadelphia, USA, for language editing of the article. The Acknowledgments section has now been removed from the article.

The original version of this article has been updated.

